# Contextual determinants on the meaning of the N word

**DOI:** 10.1186/s40064-016-2813-1

**Published:** 2016-07-20

**Authors:** Keith Allan

**Affiliations:** Monash University, Clayton, Australia; The University of Queensland, St Lucia, Australia

**Keywords:** Derogation, Context, Camaraderie, Perlocutionary effect, Perlocutionary intention

## Abstract

Use of the word *nigger* is very often castigated as slurring the referent, but this ignores the context of use. For many people the word itself is a slur no matter what the context, and such people argue for its eradication from the English language. Eradicationists confuse the form of the word with its frequent use as a slur that discredits, slights, smears, stains, besmirches people of black African descent. In this paper I discuss several occurrences of the N word in Quentin Tarantino’s film ‘Pulp Fiction’. At least one is a slur. As with many slurs, in-group usage by people who might themselves have been slurred with the term by out-groupers, *nigger* is used among African Americans to express camaraderie. Three instances of this are examined. Another instance is where black gangster millionaire Marcellus Wallace, after handing white boxer Butch Coolidge money to go down in the fifth round, tells him ‘You’re my nigger’ to which Butch replies ‘Certainly appears so’. Lastly I consider the tricky situation where a white uses the term *nigger* to a black friend, not as a term of address and not as a slur either, I argue. I discuss the composition of context and the semantics and connotations of *nigger*. I examine the place and function of the uses of *nigger* within the context of the film, ‘Pulp Fiction’, to demonstrate that the affective quality of a linguistic expression should never be judged without taking account of its intended perlocutionary effect within the context in which it is uttered. We see that the basic semantic content invariably contributes to the functional (compositional) meaning, but that pragmatic input from connotations is essential in determining the truth value of the utterance in which *nigger* appears.

## Context: its composition and importance

Context κ is (a) the world spoken of, constituted by the topic of discourse revealed by expression ε’s co-text (what is and has been said); (b) if ε is a constituent of utterance υ, such that ε ⊆ υ, κ is also the situation in which υ is expressed, which includes what is known about the speaker/writer and the perlocutionary effect of this and similar uses of ε—we might call this situation of utterance ‘the world spoken in’; finally, (c) there is a corresponding situation of interpretation in which the hearer/reader seeks to understand ε ⊆ υ (i.e. the meaning of ε in the context of the utterance in which it occurs, υ). Each ‘world’ is in fact part of a world-time pair, such that the word *world* invokes a paired time. Because worlds spoken of are revealed through language, they all have some association with the world the speaker/writer inhabits, the world spoken in.^1^ The world spoken of is a mental model of an actual or recalled or imagined world; it is a possible world accessible from the world spoken in (see Allan 2001 for more on this). A model of the world (and time) spoken of is the content of a mental space which can be readily associated in a variety of ways with other worlds (and times) occupying other mental spaces.

Allan (1981, 2011b) drew attention to the significance of salience in context to make the relevantly different interpretations of the animal nouns in sentences (1)–(6).^2^

1. It’s because Nellie likes rabbits that she won’t eat rabbit.
2. The girl holding the plate was wearing rabbit.
3. The girl who wore mink was eating rabbit.
4. Because she decided she preferred the lamb, Hetty put back the pigskin coat.
5. The butcher has some impala right now.
6. The tannery has loads of impala right now.

(1) refers to live rabbits and rabbit-meat, (2) to rabbit pelt, (3) to mink pelt and rabbit meat, (4) to lamb pelt, (5) to impala meat, and (6) to impala pelts. In (1)–(6) the different interpretations are derived from co-text, but the oddity of (8) in contrast to (7) is custom/situation-based.

(7) A. Have some more oysters.
  B. Have some more lamb [with those potatoes].
(8) ?* Have some more lambs [with those potatoes].

where one or more ingesta are normally eaten at a sitting, a countable NP is used; where only a part is normally eaten at one sitting, the uncountable (bare) form is used except in generics like *Hindus don’t eat cows, and Muslims don’t eat pigs* (b). From this follows the difference between *Have a coffee* [cup of coffee] and *Have some coffee* [from this pot]. A rather similar kind of contextual influence affects the differing interpretations of ‘old’ in (9)–(10).

(9) Queen Elizabeth II is old [uttered in 2014].
(10) Little Moreton Hall is old [uttered in 2014].

Both utterances of (9)–(10) are true as uttered in 2014: Her Majesty was born in 1926, so in 2014 she was 88 years old, which counts as old for a human; Little Moreton Hall was built very early in the 16th century, so it is approximately 500 years old, which counts as old for a building. Our knowledge of the differing life-spans of things is called upon when evaluating the particular meaning of *old* and the truth of such utterances.^3^ The time of utterance is relevant: in 1520 (9) would have been nonsense and (10) false. We are reminded of Strawson’s comment on *The King of France is wise*:

Mentioning, or referring to, something is a characteristic of a *use* of an expression, just as “being about” something, and truth-or-falsity, are characteristics of *a use* of a sentence. (Strawson 1950: 326)

As a final example in this section I adduce the much discussed (11).

(11) It’s raining.

Typically this is true if it is in fact raining at the location of the speaker/writer, though on some occasions it will be evaluated of a place known to be being spoken of as in (12):

(12) Harry’s happy it’s raining.

(12) is assumed true if it is raining wherever Harry is located (or perhaps interested in^4^) and that event makes him happy.

The primary function of language is for human beings to communicate with one another and that is achieved though utterance of spoken, written, or signed texts. All language occurs in some context or another; cf. Recanati (2005: 1): ‘sentences carry content only in the context of a speech act’. Elsewhere Recanati says that sentences ‘carry schematic meanings which only determine truth-evaluable contents in the context of a speech act’ (Recanati 2013: 61). Every utterance occurs in some time and place (a world-time pair) and it is interpreted (if at all) at a world-time pair. These provide certain aspects of context. Then there is the world and time evoked in the utterance. Take the following:

Doorbell! She stumbles through the grass barefoot, sun-huddled, drowsy. The back door leads to a poky kitchen, tiled brightly in the taste of the previous tenant. (Smith 2012: 5)

This evokes a world in which ‘she’ was barefoot outside in the back yard drowsing in the sun; there is an implicature that the doorbell rang and she went through the cramped kitchen, with its tiles she probably doesn’t like, to find out who is ringing the doorbell. The time is the present as determined by additional co-text, which will also most probably offer more information about the identity of ‘she’ and the location. Although ‘she’ could be a girl-child, the second sentence quoted makes her more likely to be adult. The reference to a previous tenant makes it most likely that the property is rented not owned. These are all things cued by the semantics of the language used but fleshed out by the pragmatic modulation of the context evoked.

A competent speaker/writer supplies sufficient context that, for the anticipated audience, a competent hearer/reader can recreate the world and time being spoken of.

## Expressions that may be used to slur

A slur is an expression of disparagement that discredits, slights, smears, stains, besmirches or sullies what it is applied to (cf. the *Oxford English Dictionary*). A slur is not, as it is often taken to be, the lexical form (or forms) in a language expression ε, but instead the perlocutionary effect of ε as a constituent of υ (such that ε ⊆ υ); the said perlocutionary effect can only be determined from κ, the context—i.e. ε’s co-text and the situations of its utterance and of its reception (see “Context: its composition and importance” section). The judgment of Anderson and Lepore (2013): 43 that ‘slurs are prohibited words; and, as such, their uses are offensive to whomever these prohibitions matter’ does not explain where such prohibitions might come from. Many people insist that lexical forms such as *nigger* invariably slur: it will be shown that *nigger*, like many other potentially offensive terms, can be used with no offense or disparagement intended or taken—a fact that is incompatible with the naïve view of the lexical form. I am not denying that the default reaction to *nigger* is that it is a slur, just as the default reaction to *shit* is that it is obscene, that the default *bird* flies^5^ and the default *bull* is bovine.^6^ The reasons for these defaults have been examined and explained in Allan (2011b, 2012, 2015a, b) and Allan and Burridge (1991, 2006). Allan (2015a) discusses an idealized benchmark for (im)politeness in Anglo communities: ‘the middle class politeness criterion’. Although defined on the ‘middle class’ as a default (much as the freezing point of water is defined as 0 °C) the criterion applies to all ranks of society; for discussion of this conundrum see Allan (2015a). (Im)politeness is never a depersonalized, decontextualized absolute but always a perception or judgment of appropriate behaviour on a given occasion; it is what one expects oneself and others to do in a particular social interaction. This ties (im)politeness to frames and scripts and to the notion of habitus (Bourdieu 1991; Eelen 2001; Mills 2003; Terkourafi 2001; Watts 2003). The middle class politeness criterion is a benchmark for behaviour, a means of managing aspects of social interaction, for which there is a counterpart in all communities. The constituents will differ in particulars for different communities but they will always identify social constraints on the use of language that are designed to maintain harmonious social relations within the community.

In order to be polite to a casual acquaintance one tends to use euphemism (*loo, bathroom; person of colour*) or orthophemism (*toilet, lavatory; African*-*American*) rather than the dispreferred dysphemism (*shithouse*; *nigger*). Orthophemisms (straight talking) and euphemisms (sweet talking) are words or phrases used as an alternative to a dispreferred (undesirable, inappropriate) expression because they avoid possible loss of face by the speaker and also the hearer or some third party. An orthophemism is typically more formal and more direct (or literal) than the corresponding more colloquial and figurative euphemism. Slurs are by definition dysphemistic, thus in order to be inoffensive, ‘politic’, and/or ‘politically correct’ (see below) in reporting a perceived slur like *nigger* a euphemism such as, in this case, *the N word* will often be used.

It is because the slur is taken to reside in the form of expression ε that the reporting of slurs is so often understood to be of itself a slur (see Allan 2016; Anderson and Lepore 2013). What I shall tease out in this essay is that racist terms like *nigger* have an affective meaning that arises from their frequent dysphemistic use in slurs and insults; consequently ε evokes strong emotions that lead to the belief that the expression in itself constitutes a slur; this is the ‘politically correct’ view and also the view of certain people who have been personally traumatized by the use of such terms (e.g. Hall 2014). Political correctness is the unwillingness to risk giving offense, especially to a group vulnerable because its members are perceived to have been mocked, disparaged, or insulted for long periods of time (see Allan and Burridge 2006 chapter 4). It might, less emotively, be described by Richard Watts’ term *politic behaviour*: ‘Politic behaviour is that behaviour, linguistic and non-linguistic, which the participants construct as being appropriate to the on-going social interaction’ (Watts 2003: 20). Any reader who thinks that the occurrence of *nigger* in this essay justifies the belief that I am slurring or insulting anyone at all is mistaken.

Although slurring must be defined on perlocutionary effect, we cannot ignore perlocutionary intention. The matter is clarified when we consider inadvertent, accidental—in other words, unintended—slurs. For instance, some readers of my essay ‘When is a slur not a slur: the use of *nigger* in “Pulp Fiction”’ (Allan 2015b) have responded that they feel slurred by the very use of the term *nigger* in the title, even though they grudgingly accept that I have no intention of using the word to slur anyone. Intentional uses of *nigger* that are non-slurs (e.g. when the expression is used to express camaraderie) should not be condemned although the speaker/writer is open to criticism by an audience member deeply offended by any use of the word. Where it is not the speaker/writer’s perlocutionary intention to be offensive but nevertheless the audience is insulted—i.e. when the perlocutionary effect of ε in υ is an accidental slur—the offense should be forgiven.

In other words, the use of *nigger* should only be condemned when the speaker/writer is recognized to have the perlocutionary intention to slur. Although a slur eventuates as a perlocutionary effect, and dysphemistic effects are properly castigated, what is more (morally) abhorrent is the intention to achieve such an effect. The speaker/writer’s intention can only be surmised from κ, the utterance context. Judging the perlocutionary effect of ε in υ as a slur is also a matter of surmise, although it is normally identifiable by the target as the sense of insult. So, both perlocutionary intention and, to a lesser degree, perlocutionary effect are open to controversy resulting from differing interpretations of the same set of data.

## The semantics and pragmatics of *nigger*

Etymologically *nigger* derives from Latin *niger* “black, dark, unlucky” which extended in late Latin to “black person”. Until the late 18th century *nig*(*g*)*er* was synonymous with *Negro*; thereafter and until the second half of the 20th century the term *nigger* was essentially a colloquial synonym for *Negro*. From earliest times until after mid-20th century the belief was prevalent—even by enlightened people such as Charles Darwin—that non-Europeans were inferior, which encouraged disparagement of them.

At some future period, not very distant as measured by centuries, the civilised races of man will almost certainly exterminate, and replace, the savage races throughout the world. At the same time the anthropomorphous apes […] will no doubt be exterminated. The break between man and his nearest allies will then be wider, for it will intervene between man in a more civilised state, as we may hope, even than the Caucasian, and some ape as low as a baboon, instead of as now between the negro or Australian and the gorilla. (Darwin 1871: 201)

Abolitionist Harriet Beecher Stowe (1811–1896) almost certainly shared a similar view to judge from her depiction of African-Americans in *Uncle Tom’s Cabin* (Stowe 1852). Social attitudes then were as distinct from those of today as people’s apparel.

In the early twentieth century there were products such as Nigger Brown Boot Polish and Nigger-Hair Tobacco where the primary purpose of the word *nigger* was descriptive rather than racist. It is unlikely that anyone involved in naming such products even considered it possible that the term could be offensive. Within the expression of the dominant social attitudes there was no more sensitivity to the feelings of minorities than there had been in Darwin’s time. Nonetheless, people gradually came to see such occurrences of *nigger* as racial slurs, albeit unintended. Attitudes have changed, and for several decades *nigger* has been widely condemned as a dysphemistic racial slur (even if condemnation by certain individuals was registered much further back in time). Dysphemism favours colloquial terms: *faeces* is not an expletive, instead we say *shit*; we tell someone to *piss off* not to *urinate off. Consequently, the colloquial term *nigger* is preferred to the more formal *Negro* as a slur. *Nigger* denotes anyone of black African descent, and is sometimes extended to other peoples of dark complexion, too.^7^

In this essay I limit discussion to the use of *nigger* in the United States of America, where as a racial slur, *nigger* is applied to African-Americans. From January 2009, America had a black President; nonetheless black males remain twice as likely to be imprisoned as Latinos and almost six times more likely than whites. White attitudes to African-Americans can be judged from the fact that both President Obama and his wife Michelle have, as black adults, been assumed by whites to be lackeys, see Westfall 2014. African-Americans are more likely than other racial groups to suffer police harassment (see, e.g. *Report of The Sentencing Project to the United Nations Human Rights Committee Regarding Racial Disparities in the United States Criminal Justice System*, August 2013 and http://sentencingproject.org). A couple of recent examples: on August 9, 2014 a white Ferguson MO police officer shot six times and killed an unarmed 18-year-old African-American male; riots ensued because of the apparent excessive force used. On November 22, 2014 in Cleveland OH a white police officer shot and killed a 12-year-old African-American boy playing with a toy pellet gun; the person who warned the police said twice that he thought the gun was fake, although this was not passed on to the officer who attended (see http://edition.cnn.com/2014/12/04/justice/cleveland-police-officer-timothy-loehmann). What such examples demonstrate is that some part of the white community has such fear of African-Americans that they are led to significantly violent overreaction which demonstrates no respect at all for the life of an African-American. This is not new, as is demonstrated by the history of racism, lynchings and less extreme mistreatments catalogued in, e.g., Asim (2007) and Kennedy (2003), and many other places. Among the negative stereotypes of African-Americans identified by Reddick (1944), Asim (2007) and Croom (2013) are that they are mentally, socially, educationally inferior; childlike, subservient, open to bad treatment, lazy, irresponsible; delinquent, menacing, inclined to crime and violence; noisy, uninhibited, sexually depraved, and licentious. If a subset of these negative stereotypical characteristics is attributed to the referent of *nigger* then the word is most probably used as a slur. In consequence, the word itself ‘evokes and provokes the underlying, almost entitled bigotry that still pervades the racial attitudes of far too many Americans, both actively and passively’ (Hall 2014).^8^ But, as I have said, *nigger* itself is innocent; the negative connotations arise from the way it is used in slurs and insults. As demonstrated at length in Allan (2015b) and as stated earlier by Kennedy (2003), Asim (2007), Coates (2013, 2014) and McWhorter (2002, 2010, 2011, 2013, 2014), inter alios, it is the context in which *nigger* is used that marks it as a slur—or not. If it is the speaker/writer’s perlocutionary intention and effect to use *nigger* in order to disparage the referent in uttering υ, then it is a slur.

Consider the situation described in an article headed ‘Fired teacher says meaning “lost in translation” after calling student “Negro”’.^9^

A Bronx teacher [Petrona Smith, 65] says her language lesson was lost in translation when she was fired for calling a student “Negro”—though she claims she was simply using the Spanish word for the color “black” at the time, according to a new lawsuit. […]

Smith, who is black and a native of the West Indies, has been unemployed since her ouster.

“They haven’t even accounted for how absurd it is for someone who’s black to be using a racial slur to a student,” said Shaun Reid, Smith’s attorney. “Talk about context! There’s a lot of things wrong here.” […]

She denied calling the student a “Negro,” and explained to investigators that she was teaching a lesson about how to say different colors in Spanish and said the word “negro,” which is Spanish for the color black. She told her students that it was not a derogatory term and that the Spanish word for a black person was “moreno.”^10^

She added that she’d been verbally abused by her charges, including being called a “f—ing monkey,” a “cockroach” and a “n—r,” but had never stooped to their level. (http://nypost.com/2013/05/23/fired-teacher-says-meaning-lost-in-translation-after-calling-student-negro)

Even if Petrona Smith had called one of her students *Negro* it is not, normally, a slur: there are fifteen occurrences of *Negro* used as a term of respect in Martin Luther King’s ‘I have a dream’ speech at the Lincoln Memorial on August 28, 1963. Furthermore, Smith herself is a Negro. And if Smith’s claim that she was simply using the Spanish term for *black* during a language lesson, then to accuse her of an offense that merited sacking is an outrageous affront to the significance of the context of her utterance and it constitutes an abuse of natural justice.

Within many minorities and oppressed groups a term of abuse used by outsiders is often reclaimed to wear as a badge of honour to mark identification with and camaraderie within the in-group. Used as an in-group term of address, *nigger* has much in common with the British and Australian address term *mate* (see Rendle-Short 2009) or American *bud*(*dy*) even though neither *bud*(*dy*) nor *mate* has the negative connotations of *nigger*. To this end, many African-Americans have adopted the term *nigger*, often respelled *nigga* (which remains homophonous), to use to or about their fellows (Allan and Burridge 1991, 2006; Asim 2007; Croom 2013; Folb 1980; Kennedy 2000, 2003; McWhorter 2002, 2010; Rahman 2012, inter alios).^11^ This is a classic example of polysemy and so although one cannot say *Ordell is a nigger*_*1*_*and so is Beaumont* [*a nigger*_*2*_] because it violates the Q-principle of both Horn (1984) and Levinson (2000), it is perfectly possible for one African-American to say to another *That honkey called me a nigger*_*2*_*, nigger*_*1*_.^12^ The speaker identifies as a person who has attracted or might attract the slur *nigger*: in other words s/he trades on the hurtful, contemptuous connotation and subverts it (cf. Hornsby 2001: 134). Examples can be found all over, e.g. in many films by Spike Lee and Quentin Tarantino. In “Eight uses of *nigger* in ‘Pulp Fiction’” section, I discuss three examples from ‘Pulp Fiction’ (1994).

## Eight uses of *nigger* in ‘Pulp Fiction’

In this essay I discuss eight of the 18 occurrences of the word *nigger* in Quentin Tarantino’s film ‘Pulp Fiction: Three stories about one story’. The narrative is composed of three distinct but interrelated storylines presented out of chronological sequence. The principal in the first story is contract killer Vincent Vega (white). Vincent’s partner is Jules Winnfield (black), and they work for gangster millionaire Marsellus Wallace (black). After liquidating some junkies who have misappropriated a briefcase belonging to Wallace, Vincent is instructed by Marsellus to entertain his wife Mia (white). The principal in the second story is prize-fighter Butch Coolidge (white) whom Marsellus pays to lose a fight; instead, Butch kills his (black) opponent. Marsellus sends Vincent to kill Butch, but the latter shoots Vincent dead with Vincent’s gun which was left in the kitchen while he used the toilet. Butch then runs Marsellus over and, recovering, Marsellus starts shooting at him. They end up captured by two hillbillies, one of whom rapes Marsellus. Butch escapes, Marsellus is freed, and the hillbillies are wounded and facing worse. The third story picks up the tale from story one, just before the murder of the junkies. Jules shoots two of the junkies, but another bursts out from a bathroom spraying bullets at the gangsters. He gets shot but no bullet has struck either Vincent or Jules, an outcome that Jules believes is a miracle and it decides him to retire from being a hit-man. Travelling away from the murder scene Vincent accidentally shoots their associate Marvin (black), whose brains spatter all over the inside of their car. To clean up the mess they visit Jules’ white friend Jimmie Dimmick where, helped by an associate of Marsellus, they clean themselves and the car up, borrow some outlandish clothes from Jimmie, and go to breakfast in a diner. The film had opened in this diner (before the credits ran) and a male–female couple of young white hoods hold it up while Vincent is in the john. Jules is determined to retire from crime and gives the hoods his money so as to buy his redemption, but he refuses to hand over Marsellus’ briefcase—the contents of which are never revealed. Jules succeeds in brow-beating the hoods and sends them on their way with their ill-gotten gains. The film ends with Jules and Vincent heading off to the action in story two.

Let me elaborate further on what I mean by ‘context’ in my discussion of ‘Pulp Fiction’. This film presents a fictional caricature of real life in Los Angeles sometime in the late 1980s, early 1990s. The cities, towns, and suburbs mentioned within the film are real locations. That’s the world of ‘Pulp Fiction’, populated by characters who are intended to simulate real people. You and I, as members of the audience, know that in the film a character may overdose on heroin, be beaten up, or killed, but that the actor playing the part does not suffer such a fate; in other words we are capable of distinguishing the world of ‘Pulp Fiction’ from the real world, even though the world of ‘Pulp Fiction’ is accessible from the real (audience) world. Social attitudes and events within the film are based on and evaluated along the same lines as events in the real world. For example, the relative social situations of blacks and whites and the unlawful acts committed within the film are meant to be judged in a similar way to these same things in real life. The same goes, of course, for the highly colloquial language used within the film, including the use of *nigger*. Although Tarantino has been criticised for using the N word in ‘Pulp Fiction’ it is because he is white, not on the basis that it does not reflect a real-life norm [see e.g. Spike Lee in *Variety* December 16, 1997, Fuchs (2002: 151), Morris O’Kelly in the *Huffington Post* January 22, 2013, Kennedy (2000, 2003) Asim (2007), McWhorter (2010, 2013, 2014), Samuel L. Jackson in Cummings (2013)]. Thus, when I refer to the film-context in my discussion of the use of *nigger* in ‘Pulp Fiction’ it is the world (and time) depicted in the film, which parodies what we know of the real world.

In story two, white hillbilly Maynard’s shop was invaded by two men fighting: Butch (white) has pinned Marsellus (black) to the floor of the pawnshop and is pointing Marsellus’ own .45 handgun in his face.

(13) Maynard (*brandishing a pump action shotgun pointed at Butch who stands above Marsellus*): Hold it right there goddammit.
  Butch: This ain’t none of your business, mister.
  Maynard: I’m making it my business. Toss the weapon.
  Butch: You don’t understand, man.
  Maynard: Toss the weapon. (*After a brief delay Butch throws the gun to his left.*) Take your foot off the nigger [1:33:2]. Put your hands behind your head. Approach the counter, right now. (*Maynard slugs Butch with the butt of his shotgun*.)

Maynard’s use of *nigger* is a definite racial slur in respect of Marsellus from the white hillbilly to the white boxer. In the prior scene where Marsellus was run over by Butch, he was assisted to his feet by a bevy of sympathetic mostly white female bystanders one of whom says ‘If you want someone to go to court, I’ll be glad to help’. This is a white woman willing to testify against a white man on behalf of an African-American—admittedly before Marsellus starts shooting at Butch and, in the process, wounding a female onlooker. Nevertheless, the contrast with the incensed pawnshop manager is stark. Consider the context: Maynard’s shop was invaded by two men fighting, so we cannot expect him to be courteous to either of them. He refers to the groggy Marsellus as ‘nigger’ and he slugs Butch with his gun. Under these circumstances the racial slur is not out of place from a dramatic point of view; whatever term was used to refer to Marsellus was going to be insulting and there are not a lot of choices: *that motherfucker* or a sarcastic *your buddy/friend* perhaps; a realistic *that cunt* would not have passed the censor; *that brother* would be inappropriate from Maynard; and *that homie/guy* would be too weak dramatically. I conclude that this occurrence of *nigger*, 93 min into the film, is a racial slur that, given the context, is dramatically justifiable and plausibly corresponds to what one could encounter in real life.

As I have said, *nigger* is used among African-Americans to express camaraderie (usually from a male to or about a male), as in (14).

(14) Jules: You remember Antwan Rockamora? Half-black, half-Samoan, usta call him Tony Rocky Horror.
  Vincent: Yeah maybe, fat right?
  Jules: I wouldn’t go so far as to call the brother fat. He’s got a weight problem. What’s the nigger gonna do, he’s Samoan. (Tarantino 1999: 18)

The context, i.e. the situation of utterance and what is said through the co-text, determines that this use of *nigger* is clearly not a racial slur. For a start Jules is black and he’s addressing a white guy while speaking of a shared acquaintance who is a half-black half-Samoan and who counts as one of Jules’ in-group of black ‘brothers’. Secondly, Jules thinks well enough of Antwan to be kindly euphemistic about his size. So when he says ‘What’s the nigger gonna do, he’s Samoan’ he is using *nigger* as a colloquial descriptive. Colloquial language uses informal and intimate styles (cf. Joos 1961); it includes, but is not identical with, slang (see Allan and Burridge 2006). So we have *nigger* used as an in-group marker, here referring to a man described as ‘Samoan’, although he is also described as ‘half-black’. Jules clearly has no malice towards this black brother of whom *nigger* is surely used in the sympathetic spirit of camaraderie.

The next example takes place in a topless bar near LAX owned by Marsellus Wallace and managed by English Dave of whom the stage direction reads: ‘*Dave isn’t really English, he’s a young black man from Baldwin Park*’. Why he’s called ‘English’ Dave is an unresolved mystery. Vincent and Jules present themselves in outlandish clothing: ‘UC Santa Cruz and “I’m with Stupid” tee-shirts, swim trunks, thongs and packing .45 automatics’ (Tarantino 1999: 187):

(15) English Dave: Vincent Vega, our man in Amsterdam. Jules Winnfield, our man in Inglewood. Git your asses on in here. Goddam, nigger, what’s up with them clothes?
  Jules: You don’t even want to know. (Tarantino 1999: 35–36)

Clearly this is an instance of banter in which an African-American is razzing a black colleague who is wearing unusual clothing (the explanation for which is not revealed to the audience until towards the end of story three; at this stage in the film we are left as ignorant as English Dave). The references to locations are not entirely insignificant: Vincent has just returned from Amsterdam—which is relevant to events in story one; and Inglewood is a dominantly black neighbourhood where Jules resides (as we learn in story three). The banter in (15) certainly reveals Dave’s disparaging view of the hit-men’s outfits but the use of *nigger* is clearly in the spirit of camaraderie and not malevolence—as we can judge from both the circumstances in which it is uttered and in Jules’ response to what Dave has said.

In story three, Jules and Vincent have shot the three white junkies who have misappropriated Marsellus’ briefcase, the contents of which are never revealed. The last of those malefactors had burst out of the bathroom firing at the gangsters but missed. Jules is convinced that ‘God came down from heaven and stopped these motherfuckin’ bullets’; Vincent is not persuaded; stool pigeon Marvin (black) is mesmerized by the horror of it all.

(16) Vincent (*to Jules*): Do you wanna continue this theological discussion in the car, or at the jailhouse with the cops?
  Jules: We should be fuckin’ dead now my friend! What happened here was a miracle, and I want you to fuckin’ acknowledge it!
  Vincent: Alright, it was a miracle. Can we go now? (*Opens the door and leaves.*)
  Jules (*to the dazed Marvin*): Let’s go nigger. [1:49:55] Come on. Shit. (*They hussle out the door*.)

The final chivvying exclamative means “get your act together” (‘Shit’ is definitely not an appellative). The context for (16) clearly reveals that Jules addressing Marvin as ‘nigger’ is in the spirit of camaraderie not insult.

The next occurrence of *nigger* that I discuss does not appear in the published script. It is uttered by Marsellus, the black gangster millionaire, to white boxer Butch as he hands Butch the bribe to go down in the fifth round of his bout with Floyd Wilson (black).

(17) Marsellus: […] How many fights d’you think you got in you anyway? Mhm? Two? Boxers don’t have an Old Timers Place. You came close but you never made it. And if you were gonna make it, you would’ve made it before now. (*Holds out the envelope of cash to Butch, but just out of his reach.*) You’re my nigger. [0:22:45]
  Butch: Certainly appears so.

Here the context (situation of utterance) offers an exquisite social irony in that a powerful African-American is calling a less powerful white man ‘my nigger’. Although *nigger* is overwhelmingly applied to blacks, to the extent that I would classify that as the core meaning of the word, in (17) this does not coerce the interpretation that Butch is black. So instead we assume, as Butch himself does, that Marsellus had some other meaning. This is not a racial slur as such, but it does play on the slur because it invokes the disparaging connotations of *nigger* as referring to an inferior, servile person—a person who carries out his master’s (or mistress’s) bidding without overt opposition. It is well-documented that *nigger* is used among African-Americans to denigrate people as well as its being, in other contexts, a term of camaraderie and banter.

The final instances of *nigger* I shall consider are uttered by a white man to an African-American about another, dead, African-American. These circumstances render the use of *nigger* especially controversial. I believe, however, that within the context identified in (18), elucidated by my backgrounding to it, there are no slurs, no insult, no deliberate disparagement. At worst, there is disrespectful colloquialism uttered without malice. The background is that while Jules is driving Vincent and Marvin back to Marsellus after the murder of the junkies, Vincent accidentally shoots Marvin whose brains spatter all over the inside of the car and its occupants. To clean up the mess they go to Jules’ friend Jimmie’s house. The three uses of *nigger* come from Jimmie, who is white.

(18) *INT. JIMMIE’S KITCHEN*—*MORNING. Vincent, Jules, and Jimmie are standing in Jimmie’s kitchen, each with a mug of coffee.*
  Jules (*drinking coffee*): Goddamn Jimmie, this is some serious gourmet shit. Me an’ Vincent woulda been satisfied with freeze-dried Tasters Choice. You spring this gourmet fuckin’ shit on us. What flavor is this?
  Jimmie (*wearing a bathrobe*): Knock it off, Julie.
  Jules: What?
  Jimmie: I’m not a cob of corn, so you can stop butterin’ me up. I don’t need you to tell me how good my coffee is. I’m the one who buys it, I know how fuckin’ good it is. When Bonnie goes shoppin’, she buys shit. I buy the gourmet expensive stuff’cause when I drink it, I wanna taste it. But what’s on my mind at this moment isn’t the coffee in my kitchen, it’s the dead nigger in my garage.
  Jules: Jimmie –
  Jimmie:—I’m talkin’. Now let me ask you a question, Jules. When you drove in here, did you notice a sign out front that said, ‘Dead nigger storage?’
  *Jules starts to ‘Jimmie’ him* –
  Jimmie: … answer the question. Did you see a sign out in front of my house that said, ‘Dead nigger storage?’
  Jules (*playing along*): Naw man, I didn’t.
  Jimmie: You know why you didn’t see that sign?
  Jules: Why?
  Jimmie:’Cause storin’ dead niggers ain’t my fuckin’ business!
  *Jules starts to ‘Jimmie’ him.*
  Jimmie: … I ain’t through! Now don’t you understand that if Bonnie comes home and finds a dead body in her house, I’m gonna get divorced. No marriage counselor, no trial separation—fuckin’ divorced. And I don’t wanna get fuckin’ divorced. The last time me an’ Bonnie talked about this shit was gonna be the last time me an’ Bonnie talked about this shit. Now I wanna help ya out Julie, I really do. But I ain’t gonna lose my wife doin’ it.
  Jules: Jimmie –
  Jimmie:—don’t fuckin’ Jimmie me, man, I can’t be Jimmied. There’s nothin’ you can say that’s gonna make me forget I love my wife. Now she’s workin’ the graveyard shift at the hospital. She’ll be comin’ home in less than an hour and a half. Make your phone calls, talk to your people, then get the fuck out of my house.
  Jules: That’s all we want. We don’t wanna fuck up your shit. We just need to call our people to bring us in. (Tarantino 1999: 146–149)

In the printed script it is never said that Bonnie is black but, about a minute following the quote above, while Jules is seeking help from Marsellus to resolve their problem, Bonnie (played by black actress Venessia Valentino) is shown in an imagined sequence:

Jules (*to Marsellus*): You got to appreciate what an explosive element this Bonnie situation is. ([1:54:47–1:55:6] *Imaginary scene where Bonnie does what Jules is hypothecating*.) If she comes home from a hard day’s work and finds a bunch of gangsters doin’ a bunch a gangsta shit in her kitchen, ain’t no tellin’ what she liable to do.^13^

Consider the various aspects of context that are relevant to understanding (18). From our perspective as audience external to the film there is the sensitive matter of a white American referring to a dead black American as a nigger. At best this is not politic (in terms of Watts 2003), not ‘politically correct’ and, potentially, it is a slur. Enlightened audience evaluation of these events will depend upon their understanding of the world depicted in the film: the personae, the criminal milieu, the events such as the accidental manslaughter, Jules’ buttering up of Jimmie, the need to dispose of the corpse and other evidence of the shooting, and so forth. The within-film context justifies me saying that what is significant in (18) is that Jimmie wants Jules, Vincent, the dead Marvin, all the gory clothes and blood-soaked car off his property before his wife returns home. It is implied that Jimmie has helped Jules out on some earlier occasion because he says: ‘The last time me an’ Bonnie talked about this shit was gonna be the last time me an’ Bonnie talked about this shit’. It’s with this on his mind that Jimmie is, understandably, upset at having a bloody car with the dead Marvin in it in his garage. He tells Jules he is apprehensive about ‘the dead nigger in my garage’, and as a white addressing a black this looks insensitive, but the situation hardly merits sensitivity. I don’t think that Jimmie is casting a slur on the dead man: he is angry about the situation. This leads to a couple more occurrences of *nigger* in one of the most frequently quoted parts of the film:

did you notice a sign out front that said, ‘Dead nigger storage?’ (*Jules starts to ‘Jimmie’ him* –) Answer the question. Did you see a sign out in front of my house that said, ‘Dead nigger storage?’ … You know why you didn’t see that sign? …’Cause storin’ dead niggers ain’t my fuckin’ business!

There is no sign in the text nor in the acting that Jules is offended. He and Jimmie are throughout using diminutives of their names, ‘Jimmie’ and ‘Julie’, which witnesses a close friendship between them. Indeed (appealing to film-external context) one cannot take a gory murdered corpse to someone’s house and ask for assistance unless there are close ties with that person.^14^ Apparently the author wants us to believe that Jimmie has the status with Jules of a ‘brother’, a white Negro, and that gives him leave to speak like a black; possibly, Bonnie’s being black is a factor here. Jules has inflicted a huge imposition on Jimmie, put Jimmie in jeopardy with the law, and for all Jimmie knows, in danger from the dead man’s friends and family. Jules has to wear Jimmie’s anger and fear which are surely contributing to his use of *nigger* in this scene. Jimmie’s use of *nigger* heightens the tension, which renders it appropriate in the context of the film at this point.

In this section of the essay I have shown that it is the context of use that determines the various nuances of meaning available for the word *nigger*. The significance of context to the proper interpretation of texts has been known for millennia and my purpose in this essay has been to deconstruct the notion of context into its component parts and elucidate the way in which the various components of context serve to determine different aspects of meaning of the same word.

## Conclusions

Use of the word *nigger* is very often castigated as slurring the referent, but this ignores the context of its use on each particular occasion. In accordance with François Recanati:

words […] are associated with are not abstract conditions of application, but rather *particular applications*. (Recanati 2005: 10)

[S]entences by themselves do not carry (even minimal) truth-evaluable contents. They carry schematic meanings which only determine truth-evaluable contents in the context of a speech act. (Recanati 2013: 61)

In the course of this study it has been shown that although the basic semantic content invariably contributes to the functional (compositional) meaning, the pragmatic input from connotations is often essential in determining the particular meaning and truth value of the utterance in which *nigger* appears.

The language of ‘Pulp Fiction’ is colloquial throughout, which explains why the term *African*-*American* never occurs. There is a single occurrence of *Negro* in the text. It is used as a term of address from Jules to Marsellus when he seeking help from Marsellus to dispose of Marvin’s corpse and the bloody car.

(19) Marsellus: You ain’t got no problems, Jules. I’m on the motherfucker. Go back in there, chill them niggers out and wait for The Wolf, who should be comin’ directly.
  Jules: You sendin’ The Wolf?
  Marsellus: Feel better?
  Jules: Shit Negro, that’s all you had to say. (Tarantino 1999: 151)

Marsellus’ use of ‘niggers’ in (19) repeats what Jules has just said to him referring to Jimmie and Vincent, both of whom are white. Arguably Jules was including himself with them. This use is reminiscent of what Marsellus said to Butch in (17) and connotes someone in powerless and perilous circumstances. At the same time they are comrades. ‘The Wolf’ is a fixer who subsequently does get all three of them out of that particular situation. The final line of the text quoted in (19) includes the only occurrence of *Negro* in the script. It’s a mark of in-group respect for the boss Marsellus who is arranging and financing the rescue; presumably addressing him as *nigger* would be too familiar in this circumstance. In ‘Pulp Fiction’ *nigger* is used of equals or inferiors and those disparaged, which reflects the norm in real life.^15^

Context κ is comprised of many components. Interpreting a text like ‘Pulp Fiction’ there is an external set of worlds and times (contexts) that contain the author, the work, and the audience.^16^ All of these potentially bear on the meaning of anything encountered within the work—as we have seen. Then there is the set of worlds and times evoked within the work itself, in this case the film ‘Pulp Fiction’. These worlds and times are revealed by the text itself, such that the meaning of a given instance of *nigger* in ‘Pulp Fiction’ is, in part at least, revealed by the co-text. Once again, all of these potentially bear on the meaning of anything encountered within the work. There is no reason to suppose that the dependence upon context for a proper understanding of the meanings in parts—or indeed, the whole—of ‘Pulp Fiction’ is not equally important for the understanding of all other language texts, whether casual conversations or legal statutes. Input from context is fundamental to the proper interpretation of any text. The latest version of Kasia Jaszczolt’s Default Semantics claims to formalise this (see Jaszczolt 2005, 2006, 2009, 2016), although I am not certain that the formalism works as claimed (see Allan 2011a). Jaszczolt (correctly) assumes that language is a socio-cultural phenomenon, formed and reformed in use, governed by the structure and operations of the brain. Consequently, words and structures bear salient, automatically retrieved, albeit sometimes irrelevant, meanings. Default Semantics is a theory of linguistic interaction that models the primary intended meaning, the most salient conveyed content whether this is achieved explicitly or implicitly. Inferential bases are flexible: a particular expression uttered in different circumstances and in different contexts can give rise to different interpretations. In this essay I have demonstrated this explicitly through the vehicle of the various meanings ascribed to the controversial word *nigger* in Quentin Tarantino’s ‘Pulp Fiction’.

## Footnotes

1 You see the effect of this if you compare, e.g., the science fiction of H. G. Wells with one of today’s SF writers.

2 See also Copestake and Briscoe (1992).

3 This parallels the different interpretations of *cut* given in Searle (1980).

4 This would have to have been identifiable from co-text.

5 Penguins and ostriches are birds.

6 Male elephants, male whales, male seals, and male alligators (among other creatures) are also bulls.

7 The relation of *nigger, nigra, nigga* to *Negro* might be compared with the similar colloquial–formal correspondences *bubby–baby, bust–burst, crick–creek, critter–creature, cuss–curse, gal–girl, hoss–horse, sassy–saucy, tit–teat*.

8 See also works such as Obama (2004) and the short stories in Packer (2003) among many others.

9 I am grateful to Pedro Chamizo Domínguez for drawing my attention to this article.

10 In fact Spanish *negro/negra* can be applied to black people.

11 There is at least one example of this in President Obama’s autobiography when, in an exchange of banter, his friend Ray addresses him as ‘nigger’, see Obama (2004: 73).

12 Assuming *nigger*_*2*_ is the slur and *nigger*_*1*_ is not.

13 Why ‘gangsters’ rather than *gangstas* or ‘gangsta’ rather than *gangster* is a mystery; as with *nigger/nigga* the difference in spelling is not matched by a difference in pronunciation.

14 Or, which is not the case here, one is in a position of much greater power.

15 Recall the fifteen occurrences of “Negro” used as a term of respect (though not of address) in Martin Luther King’s “I have a dream” speech at the Lincoln Memorial, August 28, 1963.

16 I won’t argue the case here, but I believe these components of context are always relevant to the interpretation of every utterance.
